# Efficacy and safety of Xeligekimab (GR1501) in the treatment of axial Spondyloarthritis: Results from a Phase II clinical trial

**DOI:** 10.1515/rir-2026-0016

**Published:** 2026-03-30

**Authors:** Xiaofeng Zeng, Shangzhu Zhang, Huaxiang Liu, Yi Zhao, Xiaomei Li, Zhenyu Jiang, Yu Xue, Ning Kong, Lingyun Sun, Shengyun Liu, Xiaoyun Fan, Jiankang Hu, Liying Shen, Dong Xu

**Affiliations:** Rheumatology and Immunology Department, Peking Union Medical College Hospital, Chinese Academy of Medical Sciences, Beijing, China; Department of Rheumatology, Qilu Hospital of Shandong University, Jinan, Shandong Province, China; Department of Rheumatology, Xuanwu Hospital, Capital Medical University, Beijing, China; Department of Rheumatology and Immunology, Anhui Provincial Hospital, Hefei, Anhui Province, China; Department of Rheumatology and Immunology, The First Hospital of Jilin University, Changchun, Jilin Province, China; Department of Rheumatology, Huashan Hospital, Fudan University, Shanghai, China; Department of Rheumatology and Immunology, Nanjing Drum Tower Hospital, The Affiliated Hospital of Nanjing University Medical School, Nanjing, Jiangsu Province, China; Department of Rheumatology, The First Affiliated Hospital of Zhengzhou University, Zhengzhou, Henan Province, China; Department of Rheumatology, The First Affiliated Hospital of Bengbu Medical College, Bengbu, Anhui Province, China; Department of Rheumatology and Immunology, Pingxiang People’s Hospital, Pingxiang, Jiangxi Province, China; Department of Rheumatology, Hebei Petro China Central Hospital, Langfang, Hebei Province, China

**Keywords:** Xeligekimab, axial spondyloarthritis, IL-17A, randomized controlled trial

## Abstract

**Objective:**

To evaluate the efficacy and safety of Xeligekimab (GR1501), a fully human monoclonal antibody against interleukin-17A, in Chinese patients with active axial spondyloarthritis (axSpA) who did not respond or were intolerant to nonsteroidal anti-inflammatory drugs (NSAIDs).

**Methods:**

This phase II, randomized, double-blind, placebo-controlled, multicenter study enrolled 160 patients with active axSpA. Participants were randomized equally to receive placebo or Xeligekimab at 100 mg, 200 mg, or 300 mg subcutaneously every two weeks for 16 weeks, followed by an 8-week follow-up. The primary endpoint was the Assessment of SpondyloArthritis International Society 20 (ASAS20) response at week 16. Secondary endpoints included ASAS40, ASDAS-CRP, BASDAI, BASFI, and patient-reported outcomes. Safety and immunogenicity were evaluated throughout the study.

**Results:**

At week 16, ASAS20 response rates were 52.5% with placebo and 77.5%, 75.0%, and 72.5% with Xeligekimab 100 mg, 200 mg, and 300 mg, respectively. The 100 mg and 200 mg groups showed significant improvement versus placebo (*P* < 0.05). Benefits in ASAS40, ASDAS-CRP, and BASDAI were maintained through week 24. Xeligekimab was generally well tolerated, and the overall incidence of adverse events was comparable to placebo. No treatment-emergent anti-drug antibodies were detected.

**Conclusion:**

Xeligekimab at 100 mg and 200 mg achieved meaningful clinical improvement with good tolerability in patients with active axSpA, supporting further clinical evaluation of this therapy.

## Introduction

Axial spondyloarthritis (axSpA) is a chronic inflammatory disease primarily affecting the axial skeleton, leading to pain, impaired mobility, and structural damage.^[1, 2, 3]^ It includes radiographic (AS) and non-radiographic forms,^[4]^ with a global prevalence of 0.3%–1.4% and strong HLA-B27 association.^[5,6]^ IL-17A plays a critical role in its pathogenesis.^[6]^

Current management includes nonsteroidal anti-inflammatory drugs (NSAIDs), physiotherapy, and TNF-α inhibitors.^[6, 7, 8, 9]^ While NSAIDs alleviate symptoms, they do not halt progression. TNF-α inhibitors improve outcomes but face limitations like loss of efficacy, intolerance, and infection risks.^[7,8,10]^ csDMARDs show limited efficacy for axial manifestations,^[6,11,12]^ highlighting the need for alternative therapies.

Xeligekimab (GR1501) is a fully human monoclonal antibody targeting IL-17A,^[13,14]^ a key cytokine driving inflammation and joint damage in axSpA.^[15, 16, 17]^ By neutralizing IL-17A, Xeligekimab reduces inflammation. Promising efficacy and tolerability in previous studies (*e.g*., psoriasis) support its investigation in axSpA.^[15,16,18]^

This Phase II trial aimed to evaluate the efficacy and safety of Xeligekimab in patients with active axSpA and inadequate response/intolerance to NSAIDs, providing preliminary evidence for its therapeutic potential.

## Patients and Methods

### Study Design

This randomized, double-blind, placebo-controlled, parallel-group, multicenter Phase II clinical trial was conducted across 11 clinical centers in China, with Peking Union Medical College Hospital, Chinese Academy of Medical Sciences, serving as the lead center. Patients diagnosed with axial spondyloarthritis (axSpA), including those with non-radiographic axSpA, were enrolled. The study comprised a 16-week double-blind treatment phase followed by an 8-week follow-up period, totaling 24 weeks in duration. The trial was approved by the Ethics Committee of Drug Clinical Trials, Peking Union Medical College Hospital, Chinese Academy of Medical Sciences (approval number: 2018 L02322).

### Inclusion and Exclusion Criteria

#### Inclusion Criteria

Patients diagnosed with axial spondyloarthritis, including a limited number of non-radiographic axSpA cases; Bath Ankylosing Spondylitis Disease Activity Index (BASDAI) ≥4, with a score ≥4 on question 2 of BASDAI (spinal pain); Previous inadequate response or intolerance to at least one nonsteroidal anti-inflammatory drug (NSAID).

#### Exclusion Criteria

Active Crohn’s disease or ulcerative colitis; Active tuberculosis or active infections; History of treatment with two or more TNF-alpha inhibitors or prior IL-17 inhibitors; Insufficient washout period from prior therapies; Unstable extra-articular manifestations of axSpA; Significant abnormality in hepatic, renal function, or routine blood tests; Patients with prior exposure to targeted therapies were eligible provided that protocol-defined washout periods were satisfied; however, prior IL-17 inhibitor exposure and prior use of ≥ 2 TNF inhibitors were exclusionary.

### Randomization and Blinding

#### Randomization Method

A dynamic randomization procedure was implemented using a centralized, interactive web response system (IWRS; DAS for IWRS) to ensure balanced allocation of key prognostic factors across treatment groups. A total of 160 patients were randomized in a 1:1:1:1 ratio to receive either Xeligekimab 100 mg, 200 mg, 300 mg, or placebo.

The randomization algorithm was stratified by the following three factors to minimize potential confounding: (1) Prior biologic therapy for axial spondyloarthritis (axSpA): Naïve; Experienced (including but not limited to prior exposure to adalimumab, etanercept, *etc*.). (2) Disease severity: Based on baseline high-sensitivity C-reactive protein (hs-CRP) level: ≥ 10 mg/L; < 10 mg/L. (3) axSpA Classification: Non-radiographic axial spondyloarthritis (nr-axSpA); AS.

The IWRS dynamically assigned patients to treatment groups, balancing the distribution of these stratification factors across all four arms. Electronic emergency unblinding envelopes were provisioned within the system for use only in medical emergencies. Enrollment was conducted competitively across all participating clinical centers.

### Study Assessments and Visit Schedule

Patients were evaluated at screening, baseline and at scheduled follow-up visits at Weeks 2, 4, 8, 12, 16, 20, and 24. A comprehensive set of efficacy, safety, pharmacokinetic (PK), immunogenicity, and patient-reported outcome (PRO) measures were collected throughout the study.

#### Blood Sample Collection

Blood samples were collected at specified time points for various analyses. PK samples were drawn to determine plasma concentrations of Xeligekimab for the calculation of parameters. Immunogenicity samples for detecting anti-drug antibodies (ADAs) were collected at Weeks 0 (Baseline), 2, 4, 16, and 24. Exploratory biomarker samples for peripheral blood IL-17A levels were measured at Weeks 0, 4, and 16. Additionally, routine laboratory safety tests (hematology, blood chemistry, urinalysis, and coagulation function) were performed at each scheduled visit to monitor patient safety.

#### Assessments Performed at Each Visit

At every study visit (Weeks 2, 4, 8, 12, 16, 20, and 24), the following core set of assessments was performed: recording of adverse events (AEs), physical examination, vital signs, laboratory safety tests, and 12-lead electrocardiogram (ECG). Patient-reported outcomes, including the Patient Global Assessment of Disease Activity and the Investigator Global Assessment of Disease Activity using Numerical Rating Scales (NRS), were also collected at each visit.

The primary efficacy endpoint, the proportion of patients achieving an Assessment of Spondyloarthritis International Society 20 (ASAS20) response, was assessed at Week 16. Secondary efficacy endpoints were evaluated at multiple time points. ASAS20 and ASAS40 responses were assessed at Weeks 2, 4, 8, 12, 16, 20, and 24. ASAS partial remission and ASAS5/6 responses were assessed at Weeks 12, 16, and 24. Disease activity scores, including the Ankylosing Spondylitis Disease Activity Score (ASDAS-CRP and ASDAS-ESR) and the BASDAI, were evaluated at Weeks 2, 4, 8, 12, 16, 20, and 24. Functional status, as measured by the Bath Ankylosing Spondylitis Functional Index (BASFI), was also assessed at all visits from Week 2 to Week 24. Spinal mobility, assessed by the Bath Ankylosing Spondylitis Metrology Index (BASMI), was measured at Weeks 12, 16, and 24. Enthesitis (using the Maastricht Ankylosing Spondylitis Enthesitis Score, MASES) and inflammation on MRI (using the Spondyloarthritis Research Consortium of Canada [SPARCC] score) were assessed at Weeks 16 and 24.

Additional patient-reported outcomes, including the Columbia-Suicide Severity Rating Scale (C-SSRS), Work Productivity and Activity Impairment (WPAI) questionnaire, Functional Assessment of Chronic Illness Therapy (FACIT) scale, and Ankylosing Spondylitis Quality of Life (ASQoL) questionnaire, were administered at Baseline (Week 0), Week 16, and Week 24.

This structured schedule ensured comprehensive and systematic data collection for all pre-specified study endpoints throughout the 24-week trial period.

### Study Intervention

In this study, participants were randomized into four parallel groups to receive either placebo or different doses of Xeligekimab (100 mg, 200 mg, or 300 mg). The study drug or placebo was administered subcutaneously once every two weeks. The total treatment duration was 16 weeks, followed by an 8-week follow-up period without treatment. Dosage selection for each group was based on previous pharmacokinetic and safety data from earlier-phase studies. The placebo group received placebo injections matched in appearance and administration schedule to the active treatment to maintain blinding throughout the study duration.

### Concomitant Medications

All prior and concomitant medications (including prescription and over-the-counter drugs) used by enrolled subjects from at least 30 days before screening until the end of the follow-up period were recorded in the source documents and electronic case report forms (eCRFs). Details recorded included the therapeutic purpose, start and end dates, dosage, frequency, and route of administration. Any changes in dosage, frequency, or administration during the trial were also documented.

#### Permitted Medications and Stability Requirements

During the study, patients were permitted to continue stable background therapies for axial spondyloarthritis (axSpA) under specific conditions:

NSAIDs and Corticosteroids: Stable doses of NSAIDs and corticosteroids (at a dose ≤10 mg/d prednisone equivalent) were allowed from 2 weeks prior to randomization until the end of the study.

Conventional Disease-Modifying Antirheumatic Drugs (cD-MARDs): Patients receiving methotrexate (MTX; ≤ 25 mg/wk), sulfasalazine (≤ 3 g/d), or hydroxychloroquine (≤ 400 mg/d) were eligible if the treatment had been ongoing for more than 3 months and the dose had been stable for at least 4 weeks prior to randomization.

For patients taking MTX, concomitant administration of folic acid (approximately 5 mg per week) was mandatory, typically administered within 48 h after the MTX dose (but not on the same day as MTX).

#### Dosing Restrictions

During the initial 16-week, placebo-controlled phase of the trial, the doses of these permitted background medications (including NSAIDs and corticosteroids) were not to be changed, and no new corticosteroid or NSAID therapy was to be initiated. After Week 16, adjustments to background therapy were permitted at the investigator’s discretion.

#### Prohibited Medications

The following medications were strictly prohibited during the study period: (1) Leflunomide and other cDMARDs not specified above (*e.g*., thalidomide, iguratimod), as well as Chinese herbal medicines or formulations (*e.g*., Tripterygium wilfordii) used for axSpA treatment;

(2) Any biologic therapy affecting axSpA, including but not limited to etanercept, infliximab, golimumab, abatacept, anakinra, rituximab, secukinumab, and their biosimilars;

(3) Opioid analgesics (with the exception of tramadol);

(4) Intra-articular, spinal, or para-spinal injections of corticosteroids.

### Study Outcomes

The primary efficacy outcome was the proportion of patients achieving the ASAS20 response at week 16. ASAS20 response was defined as improvement of ≥ 20% and absolute improvement of ≥1 unit (on a 0–10 scale) in at least three of the four ASAS domains (patient global assessment, pain, function, and inflammation), with no worsening in the remaining domain. Secondary efficacy outcomes included ASAS40 response, defined as ≥ 40% improvement and ≥2 units absolute improvement, ASAS partial remission, ASAS 5/6 response, and various disease activity measures such as ASDAS-CRP, ASDAS-ESR, BASDAI, BASFI, BASMI, MASES, and SPARCC. Changes from baseline in these indices were assessed at predetermined intervals throughout the 24-week study duration. Safety outcomes included the incidence of AEs, serious adverse events (SAEs) and immunogenicity assessments.

### Sample Size Determination

According to the pre-specified study protocol, a total of 160 patients were planned for enrollment in this trial. Following a 2-week screening period, eligible patients were to be randomized in a 1:1:1:1 ratio into one of four treatment groups: the 100 mg group, the 200 mg group, the 300 mg group, or the placebo group.

### Patient Registration and Randomization

Patient enrollment was conducted across multiple clinical centers. Eligible patients who successfully completed the 2-week screening period were centrally registered and randomized *via* an Interactive Web Response System (IWRS; DAS for IWRS). The system automatically assigned a unique randomization number and allocated patients in a 1:1:1:1 ratio to the 100 mg, 200 mg, 300 mg, or placebo group. All centers enrolled patients continuously without predefined site-specific quotas until the total target sample size of 160 was reached. The IWRS ensured consistent and blinded treatment allocation across all study sites.

### Statistical Analysis

Efficacy analyses were conducted on the full analysis set (FAS), defined as all randomized participants who received at least one dose of study medication and had at least one efficacy assessment. The primary efficacy endpoint was the proportion of patients achieving ASAS20 at Week 16. Statistical comparisons between treatment and placebo groups were performed using the Chi-square test for categorical variables and t-tests for continuous variables. A two-sided *P*-value < 0.05 was considered statistically significant. Continuous data were expressed as means ± standard deviations (SD), while categorical data were presented as proportions (%). Secondary endpoints and safety analyses were presented descriptively.

## Results

### Baseline Characteristics of Patients

**Figure 1:**
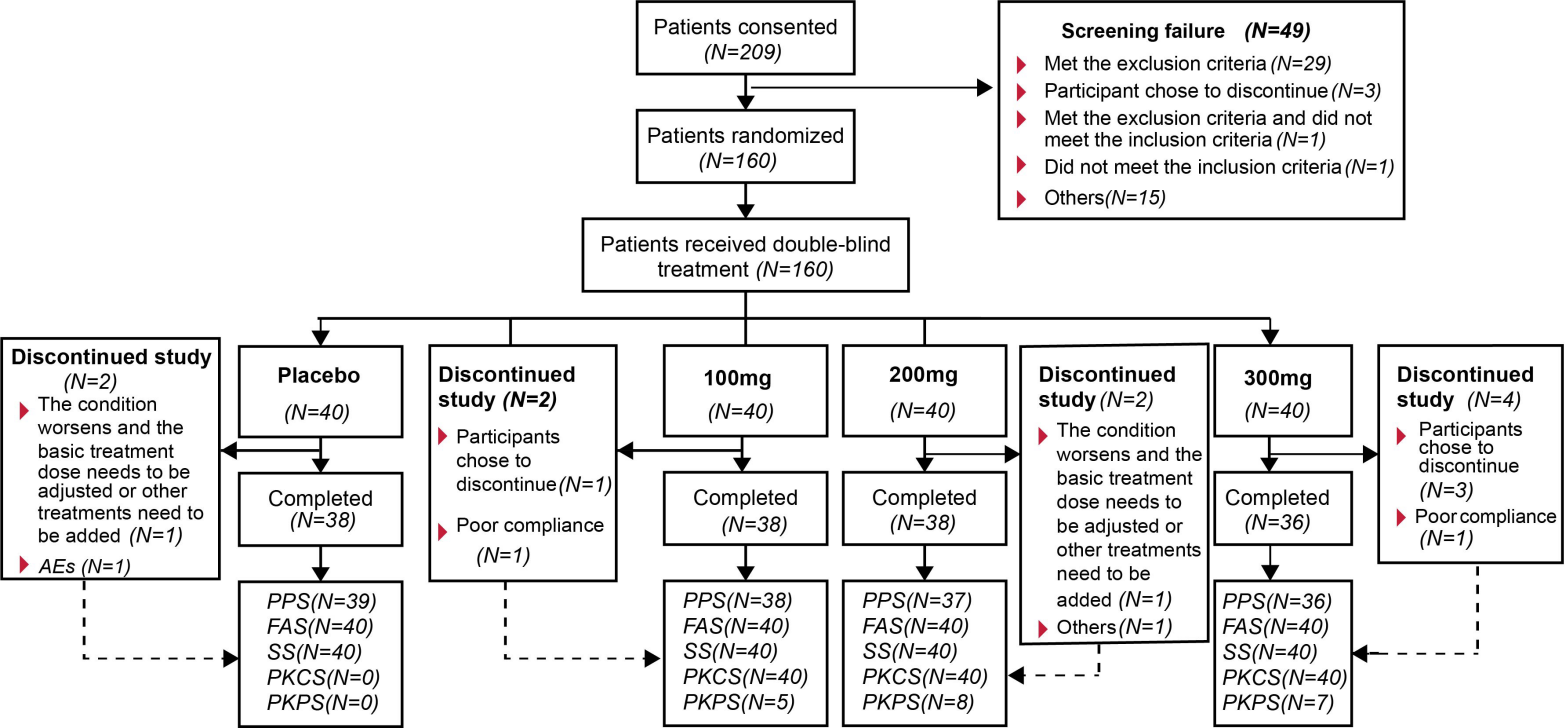
Patient flowchat. PPS, per-protocol set; FAS, full analysis set; SS, safety set; PKCS, pharmacokinetic concentration set; PKPS, pharmacokinetic parameter set; TEAE, treatment-emergent adverse event; ADR, adverse drug reaction; AE, adverse event.

A total of 160 patients were enrolled and randomized equally (1:1:1:1) into placebo, Xeligekimab 100 mg, 200 mg, and 300 mg groups (40 patients per group). All 160 randomized patients were included in the FAS for efficacy and the safety analysis set (SS). The per-protocol set (PPS) consisted of 150 patients who completed the study without major protocol deviations. Baseline characteristics including demographics, disease activity (BASDAI ≥ 4 with spinal pain ≥ 4), and prior treatment experiences were well-balanced among all groups. (Figure 1). Baseline characteristics were comparable between the treatment and placebo groups (Table 1).

**Table 1:** Demographics and baseline clinical characteristics of Chinese participants with Axial Spondyloarthritis in a phase II trial of xeligekimab (GR1501)

Parameters	Placebo (*N* = 40)	100 mg (*N* = 40)	200 mg (*N* = 40)	300 mg (*N* = 40)	Total (*N* = 160)	Statistic	*P*-value	Method
Age (yr)	33.3 ± 8.91	32.6 ± 6.26	35.3 ± 8.64	35.1 ± 8.24	34.1 ± 8.09	1.090	0.355	ANOVA
Height (cm)	169.03 ± 9.792	172.51 ± 7.820	170.55 ± 8.693	168.36 ± 7.689	170.11 ± 8.20	2.062	0.107	ANOVA
Weight (kg)	69.72 ± 13.161	76.19 ± 14.139	71.15 ± 13.579	71.33 ± 13.689	72.09 ± 13.736	1.713	0.167	ANOVA
BMI (kg/m^2^)	24.45 ± 4.349	25.54 ± 4.052	24.40 ± 4.019	25.14 ± 4.417	24.88 ± 4.201	0.693	0.557	ANOVA
Gender (Male *n* [%])	33 (82.5)	38 (95.0)	38 (95.0)	35 (87.5)	144 (90.0)	4.969	0.174	Chi-square R*C
Gender (Female *n* [%])	7 (17.5)	2 (5.0)	2 (5.0)	5 (12.5)	16 (10.0)			
Ethnicity (Han *n* [%])	37 (92.5)	39 (97.5)	37 (92.5)	39 (97.5)	152 (95.0)	2.092	0.554	Chi-square R*C
Ethnicity (Others *n* [%])	3 (7.5)	1 (2.5)	3 (7.5)	1 (2.5)	8 (5.0)			
Radiographic axial spondyloarthritis	3 (7.5)	3 (7.5)	3 (7.5)	2 (5.0)	11(6.9)	0.291	0.962	Chi-square R*C
Ankylosing spondylitis	37 (92.5)	37 (92.5)	37 (92.5)	38 (95.0)	149(93.1)			
History of Prior Biologic Therapy for Axial Spondyloarthritis						1.766	0.622	Chisq R*C
No, *n* (%)	36(90.0)	32(80.0)	35(87.5)	34(85.0)	137(85.6)			
Yes, *n* (%)	4(10.0)	8(20.0)	5(12.5)	6(15.0)	23(14.4)			

GR1501, xeligekimab.

### Efficacy Evaluation

#### ASAS20 and ASAS40 Response Rates

**Figure 2:**
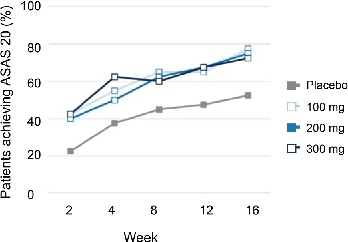
ASAS20 response rate in each dose group at week 16. ASAS20, assessment of SpondyloArthritis international society 20.

**Figure 3:**
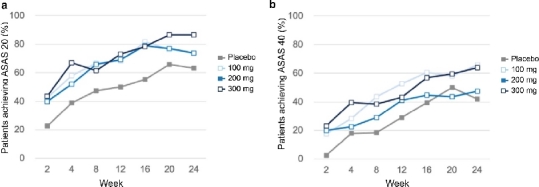
ASAS20 and ASAS40 response rates at week 24. (a) ASAS20 response rate in each dose group at week 24; (b) ASAS40 response rate in each dose group at week 24. ASAS20, assessment of SpondyloArthritis international society 20.

At week 16, the proportion of patients achieving ASAS20 response in placebo, Xeligekimab 100 mg, 200 mg, and 300 mg groups were 52.5%, 77.5%, 75.0%, and 72.5%, respectively. Both the 100 mg and 200 mg dose groups showed statistically significant improvements compared to placebo (Figure 2, *P* < 0.05). The 300 mg group showed numerically greater improvement compared to placebo; however, the difference did not reach statistical significance (*P* > 0.05). By week 24, ASAS20 response rates increased further to 63.2% in placebo, and 73.7%, 73.7%, and 86.1% in the 100 mg, 200 mg, and 300 mg groups, respectively. ASAS40 response rates at week 24 were 42.1% (placebo), 65.8% (100 mg), 47.4% (200 mg), and 63.9% (300 mg), indicating sustained efficacy for Xeligekimab (Figure 3). The between-group differences at Week 24 were statistically significant for the 100 mg group versus placebo (*P* < 0.05), while no significant differences were observed for the other dose groups at any time point (*P* > 0.05).

#### Improvement in Disease Activity Scores

**Figure 4:**
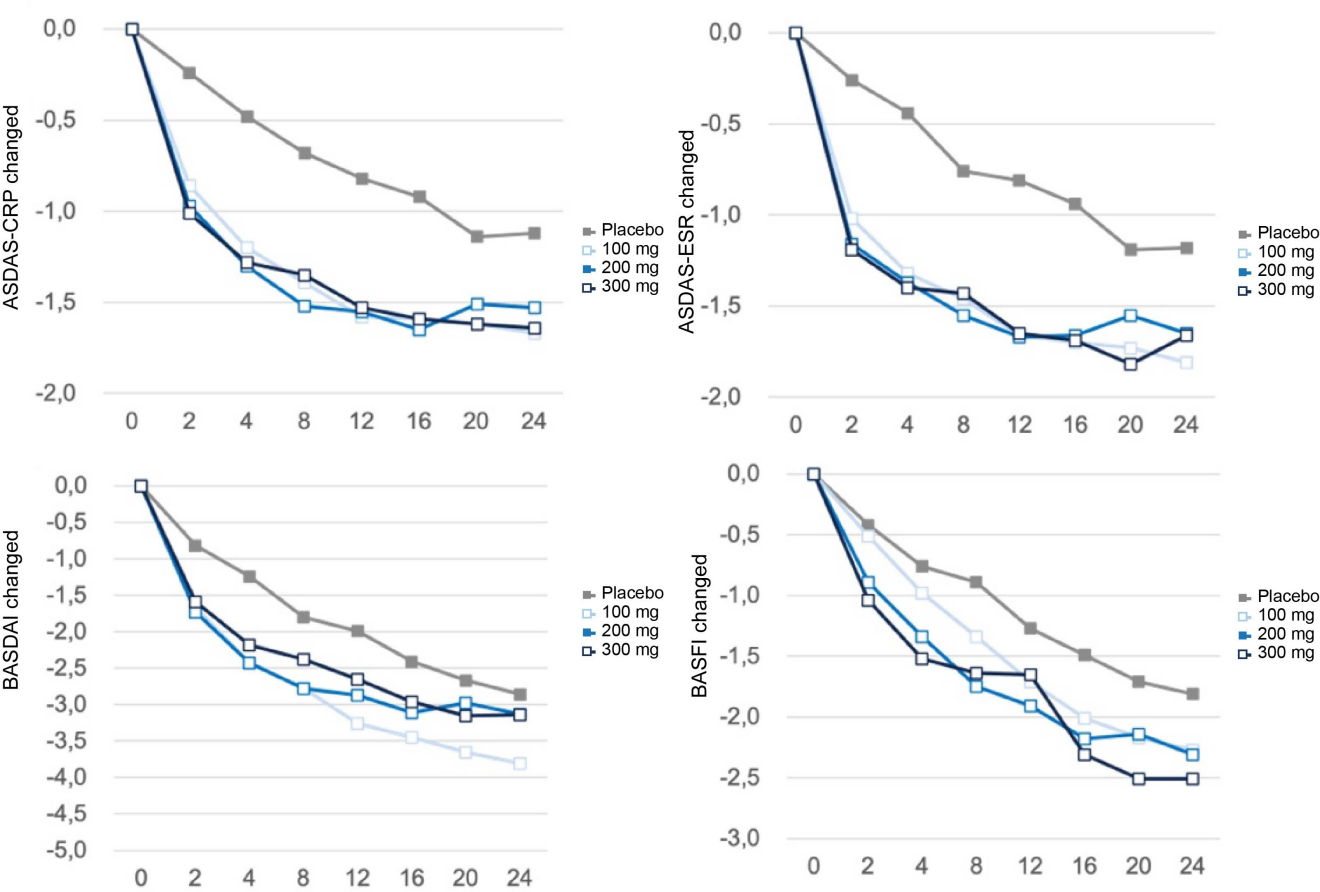
Changes in ASDAS-CRP, ASDAS-ESR, BASDAI, and BASFI over 24 weeks. (a) A scoring system that combines CRP and clinical symptoms to measure disease activity (ASDAS-CRP); (b) An index that combines ESR and clinical symptoms to assess disease activity (ASDAS-ESR); (c) An index that assesses disease activity based on 6 symptoms (such as fatigue, pain, and stiffness) (BASDAI); (d) A scoring tool that assesses the degree of functional limitation of patients through 10 questions about daily activities (BASFI). CRP, C-reactive protein; SDAS, ankylosing spondylitis disease activity score; ESR, erythrocyte sedimentation rate; BASDAI, bath ankylosing spondylitis disease activity index; BASFI, bath ankylosing spondylitis functional index.

All active treatment groups showed significant reductions from baseline in ASDAS-CRP, ASDAS-ESR, BASDAI, and BASFI scores compared with placebo across multiple time points up to week 24. Improvements were noted as early as week 2 and continued progressively throughout the study. The active treatment groups consistently exhibited superior disease control, with mean scores below 4 points achieved from week 4 onward, indicating substantial clinical improvement (Figure 4).

#### Patient-reported Outcomes (Pros)

**Figure 5:**
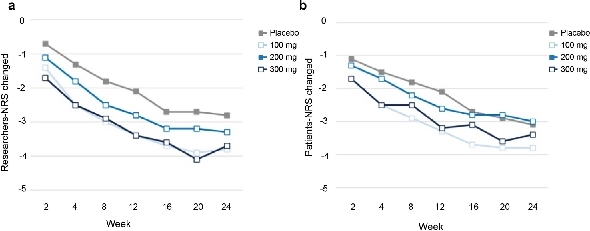
Changes in NRS scores over time: (a) researcher-rated and (b) patient-rated. (a) Researcher’s NRS score change over time and (b) Patient’s NRS score change over time. NRS, numerical rating scales.

Both Investigator and patient NRS scores showed progressive improvements across all dose groups, significantly exceeding placebo from week 8 to week 24. At the end of week 24, mean NRS scores in the treatment groups were consistently below 4 points, suggesting a clinically meaningful reduction in pain and symptom severity reported by patients (Figure 5).

### Safety Evaluation

#### Adverse Events (Ae) and Serious Adverse Events (SAe)

Adverse event rates were slightly higher in the 300 mg group compared to the 100 mg, 200 mg, and placebo groups. The most commonly reported AEs (incidence ≥ 5%) were upper respiratory tract infections, injection-site reactions, oral ulcers, and abdominal pain. No new or unexpected safety concerns were identified (Table 2). The incidences of treatment-emergent adverse events (ADR s) were 87.5% (100 mg), 77.5% (200 mg), 90.0% (300 mg), and 77.5% (placebo). The proportions of adverse drug reactions (ADR) were 60.0% (100 mg), 72.5% (200 mg), and 52.5% (placebo). The higher incidence of adverse events observed in the 300 mg group, however, there is no linear relationship between the side effects. But suggested that doses of 100–200 mg may be preferable for subsequent clinical trials.

**Table 2:** Adverse events recorded in Chinese patients with Axial Spondyloarthritis during the induction dosing periods of a phase II trial of GR1501

Parameters	100 mg (*N* = 40)	200 mg (*N* = 40)	300 mg (*N* = 40)	Placebo (*N* = 40)
TEAE, %	87.5%	77.5%	90.0%	77.5%
ADR, %	60.0%	60.0%	72.5%	52.5%
AEs occurring ≥ 5%				
Upper respiratory infection	17.5%	12.5%	30.0%	22.2%
Injection site reaction	10.0%	20.0%	12.5%	7.5%
Oral ulcer	7.5%	5.0%	5.0%	2.5%
Hyperlipidemia	20.0%	12.5%	7.5%	7.5%
Toothache	0	0	0	5.0%
Diarrhea	2.5%	0	10.0%	5.0%
Abdominal discomfort	5.0%	5.0%	3.0%	5.0%
ADR leading to death, *n* (%)	0 (0)	0 (0)	0 (0)	0 (0)
TEAE leading to discontinuation, *n* (%)	0 (0)	0 (0)	0 (0)	1 (2.5)
ADR leading to discontinuation, *n* (%)	0 (0)	0 (0)	0 (0)	1 (2.5)

TEAE, treatment-emergent adverse events; ADR, adverse drug reactions; AEs, adverse events; GR1501, xeligekimab.

#### Immunogenicity

The immunogenicity results showed that no subject in the 100 mg, 200 mg, and 300 mg groups had a negative result before medication and then a positive result after medication. After 4 wk and 16 wk of treatment, the measured values ​of IL-17A levels in the 100 mg, 200 mg, 300 mg, and placebo groups were significantly increased compared to the baseline, and the IL-17A levels of the three groups of the study drug at 4wk and 16wk after treatment were much higher than those in the placebo group.

## Discussion

### Clinical Value of Xeligekimab in the Treatment of Axial Spondyloarthritis

This phase II clinical trial revealed that Xeligekimab (GR1501), a humanized monoclonal antibody that specifically targets interleukin-17A (IL-17A), has substantial clinical potential for the treatment of axial spondyloarthritis (axSpA). At week 16, the response rates of ASAS20 in the 100 mg and 200 mg groups were substantially higher than those of the placebo (52.5%), with 77.5% and 75.0%, respectively. This highlights the therapeutic potential of this agent in reducing the activity of axSpA disease. These results are consistent with the previous evidence from clinical studies involving other IL-17A inhibitors, such as secukinumab and ixekizumab,^[19, 20, 21]^ and support the role of IL-17A inhibition as a valuable therapeutic strategy in axSpA.^[20,21]^

Furthermore, the continuous enhancements noted through week 24 across many endpoints—including ASAS40 responses, ASDAS-CRP, BASDAI, and BASFI—underscore the prospective efficacy of Xeligekimab. The results demonstrate that specific suppression of IL-17A can significantly reduce inflammation and functional impairment related to axSpA, thereby reinforcing IL-17A as a vital therapeutic target.^[22, 23, 24]^

### Clinical Implications of the Study Results

The findings from this study have significant implications for the treatment of axSpA. The shown efficacy of Xeligekimab at 100 mg and 200 mg doses in attaining ASAS20 and ASAS40 responses, coupled with significant reductions in BASDAI, BASFI, and ASDAS-CRP scores, substantiates these doses as ideal treatment options, effectively balancing efficacy and tolerability. The 100–200 mg dosage range is particularly significant, since it yielded similar therapeutic results with a reduced occurrence of side events compared to the higher 300 mg dosage group. This discovery corresponds with the clinical objective of attaining optimal efficacy while reducing treatment-related risks.^[22,25]^ Xeligekimab demonstrated a numerically higher response rate compared to both secukinumab (56%) and ixekizumab (59.5%). This suggests a potentially favorable efficacy profile for Xeligekimab.

Significant patient-reported outcomes, demonstrated by marked enhancements in NRS scores from both patients and investigators, indicate that Xeligekimab effectively diminishes disease activity while considerably enhancing patient quality of life and functional capacity.^[20,22]^ Thus, Xeligekimab may serve as a significant therapeutic alternative for individuals exhibiting insufficient response or intolerance to existing standard treatments, thereby broadening clinical options and enhancing personalized patient management.^[26,27]^

### Limitations of the Study

Despite these encouraging outcomes, some restrictions warrant consideration. The limited sample size (160 patients) and brief trial length (24 weeks) hinder our capacity to comprehensively assess the long-term efficacy, safety, and possible structural modification effects of Xeligekimab. Longer-duration studies with bigger populations are necessary to validate the current findings and establish long-term safety profiles.

Furthermore, this trial did not include complete imaging outcomes, such as radiographic or MRI evaluations, so constraining our comprehension of Xeligekimab’s effect on disease structural development. Future research utilizing improved imaging techniques would elucidate if enhancements in clinical results correspond to the suppression of structural progression.^[28,29]^

At some point while the trial sample comprised Chinese axSpA patients exhibiting poor response or intolerance to NSAIDs, the applicability of the findings to wider global populations with varied genetic backgrounds and illness severities requires further investigation.^[30,31]^

Finally, this study has two notable limitations. First, data on prior exposure to tumor necrosis factor inhibitors (TNFi) were not systematically collected, precluding a subgroup analysis of efficacy based on TNFi treatment history. Second, the immunogenicity profile of Xeligekimab could not be fully characterized due to the lack of anti-drug antibody (ADA) assessment; the reported serum IL-17A levels primarily serve as a pharmacodynamic marker of target engagement rather than a measure of immunogenicity.

## Conclusion

In this phase II randomized, double-blind, placebo-controlled trial in Chinese patients with active axSpA and inadequate response or intolerance to NSAIDs, Xeligekimab demonstrated clinically meaningful improvements in key efficacy outcomes, with significant ASAS20 responses at Week 16 for the 100 mg and 200 mg doses versus placebo and sustained benefits through Week 24. Overall, Xeligekimab showed a safety profile comparable to placebo with no treatment-emergent anti-drug antibodies observed. These findings support further clinical development of Xeligekimab, with larger and longer-term studies warranted to confirm efficacy, define optimal dosing, and evaluate long-term safety and structural outcomes.
